# Multiple independent structural dynamic events in the evolution of snake mitochondrial genomes

**DOI:** 10.1186/s12864-018-4717-7

**Published:** 2018-05-10

**Authors:** Lifu Qian, Hui Wang, Jie Yan, Tao Pan, Shanqun Jiang, Dingqi Rao, Baowei Zhang

**Affiliations:** 1 Anhui Key Laboratory of Eco-engineering and Bio-technique, School of Life Sciences, Anhui University, Hefei, 230601 China; 2 Kunming Institute of Zoology, Chinese Academy of Sciences, Kunming, 650223 China; 3 Jiangsu Key Laboratory for Biodiversity and Biotechnology, College of Life Sciences, Nanjing Normal University, Nanjing, 210046 China

**Keywords:** Mitochondrial genome, Gene rearrangement, Hotspots, Duplicate control regions, Snakes

## Abstract

**Background:**

Mitochondrial DNA sequences have long been used in phylogenetic studies. However, little attention has been paid to the changes in gene arrangement patterns in the snake’s mitogenome. Here, we analyzed the complete mitogenome sequences and structures of 65 snake species from 14 families and examined their structural patterns, organization and evolution. Our purpose was to further investigate the evolutionary implications and possible rearrangement mechanisms of the mitogenome within snakes.

**Results:**

In total, eleven types of mitochondrial gene arrangement patterns were detected (Type I, II, III, III-A, III-B, III-B1, III-C, III-D, III-E, III-F, III-G), with mitochondrial genome rearrangements being a major trend in snakes, especially in Alethinophidia. In snake mitogenomes, the rearrangements mainly involved three processes, gene loss, translocation and duplication. Within Scolecophidia, the O_L_ was lost several times in Typhlopidae and Leptotyphlopidae, but persisted as a plesiomorphy in the Alethinophidia. Duplication of the control region and translocation of the tRNA^Leu^ gene are two visible features in Alethinophidian mitochondrial genomes. Independently and stochastically, the duplication of pseudo-Pro (P*) emerged in seven different lineages of unequal size in three families, indicating that the presence of P* was a polytopic event in the mitogenome.

**Conclusions:**

The *WANCY* tRNA gene cluster and the control regions and their adjacent segments were hotspots for mitogenome rearrangement. Maintenance of duplicate control regions may be the source for snake mitogenome structural diversity.

**Electronic supplementary material:**

The online version of this article (10.1186/s12864-018-4717-7) contains supplementary material, which is available to authorized users.

## Background

In general, mitochondrial genomes (mitogenomes) of vertebrates are double-stranded circular molecules, typically 16-18 kbp in size and encode a set of 37 genes, including 2 ribosomal RNA genes, 22 tRNA genes and 13 respiratory protein genes [1–7]. Vertebrate mitochondrial genomes also contain a control region (CR), which include signals for the initiation of replication and transcription [8, 9]. A short non-coding replication origin for the L-strand (O_L_) also has been identified in the mitochondrial genomes of most vertebrates, excluding birds, crocodilians, tuatara and blind snakes [10, 11]. Compared with nuclear DNA, the evolution of mitogenome sequences is rapid, they lack introns, are highly conserved in gene content and order [12], and are abundant in cells. These special features make them valuable for studying organismal evolution, phylogeny and taxonomy [7, 12].

Generally, the organization of the 37 genes and the major noncoding regions (control region, CR) tend to be conserved in vertebrates [5, 6, 9]. However, deviations from the typical organization pattern have been found in many animal groups, such as fish [13, 14], amphibians [3, 5], reptiles [6, 10, 15–19], birds [7, 20], and mammals [21, 22]. Such deviations involve shuffling of tRNA gene clusters, translocations and/or duplications of genes, loss of genes, and some gene inversions [3, 5–7, 13, 20, 23–25]. Gene rearrangements in vertebrate mitogenomes can be explained using two widely accepted models, the Tandem Duplication and Random Loss (TDRL) Model [26] and the Recombination Model [27]. The TDRL model was postulated to account for most vertebrate gene rearrangements; it posited that rearrangements of mitochondrial gene order have occurred via tandem duplication of some genes, followed by the random deletion of some of the duplications [6, 13, 14, 20, 26, 28–30]. The recombination model is characterized by breakage and rejoining of the participating DNA strands, and has often been used to explain changes in mitochondrial gene order [14, 31–35]. There are also two further models, Tandem Duplication and Non-Random Loss (TDNL) model, and tRNA mis-priming, which are less commonly used to explain mitogenome rearrangements [36–38]. The TDNL model assumes a complete mtDNA duplication followed by the loss of genes, predetermined by their transcriptional polarity and location in the genome; the tRNA mis-priming model considers the duplicated/inserted tRNAs acting as primers for DNA synthesis, but these tRNA primer sequences fail to be removed from the nascent DNA strand during mtDNA replication.

The mitogenomes of snakes contain a number of characteristics that are unusual for vertebrates and represent an ideal model for exploring potential links between mitogenomic structure, function, and evolution [25]. These unique characteristics are the duplicated control regions, an elevated evolutionary rate relative to nuclear DNA. and shorter tRNA genes, and other shortened genes [12, 25, 39, 40].

In 1998, the first reported snake mitogenome came from a Japanese colubrid snake, the akamata (*Lycodon semicarinatus*) [41], whose gene order differed from most known vertebrates at that time owing to its duplicated control regions (Additional file 1: Figure S1). By 2005 (Additional file 2: Table S2) only 10 snake mitogenomes had been reported with new mitogenome arrangements and genomic characteristics having been discovered [10, 16, 17, 41, 42]. The mitogenomic arrangement in the Texas blind snake (*Leptotyphlops dulcis*) is distinct from alethinophidian snakes by having a single control region. In addition, the light-strand replication origin (O_L_) was lost in the Texas blind snake, and the tRNA^Gln^ gene translocated from *IQM* to the *WANCY* cluster (Additional file 1: Figure S1B). The duplicated control regions were the most important feature in alethinophidian snakes. It has been found that duplicated control regions evolved concertedly and were accompanied by the tRNA^Leu^ gene translocation [18]. Another feature new mitogenome arrangements was the appearance of pseudo-Pro (P*) in *L. semicarinatus* (Colubridae) and *Ovophis okinavensis* (Viperidae). But the P* was located at the 5′ proximity of CR2 in *L. semicarinatus* whilst at the 5′ proximity of CR1 in *O. okinavensis* (Additional file 1: Figure S1C, D, E) [3, 10, 16]. Yan et al. [18] compared the mitogenomes of 14 snake species from 11 families and found six distinct gene arrangement patterns (Additional file 1: Figure S1). They believed that the mitogenome of the brahminy blind snake *Ramphotyphlops braminus* (Typhlopidae) was the ancestral arrangement (Additional file 1: Figure S1A). The P*, located at the 5′ proximity of CR2, was viewed as a unique characteristic feature of the Families Colubridae and Homalopsidae. However, the P* could have originated from two different evolutionary events independently in the families Colubridae and Homalopsidae, (Additional file 1: Figure S1) [18]. In 2009, a new mitogenome arrangement was found in the king cobra *Ophiophagus hannah* (Elapidae) (Additional file 1: Figure S1G) [19]. In the new mitogenome arrangement, the tRNA^Ile^ was duplicated from the 3′ proximity of NADH dehydrogenase 1 (*ND1*) and inserted into the 5′ proximity of the CR1. Apart from the above mitogenome arrangement patterns, three new patterns were discovered in Colubridae [43, 44]. In the blunt-headed tree snake (*Imantodes cenchoa*), the mitogenome arrangement was marked by unequal sized duplicate control regions; the second arrangement, found in small spotted cat-eyed snake *Leptodeira septentrionalis*, was characterized by unequal size duplicate control regions accompanied by a non-coding fragment insertion between *ND5* and *ND6*; and the last arrangement, contained a large (5702 bp) insertion between tRNA^Cys^ and tRNA^Tyr^ and was found in the clouded snake *Sibon nebulatus* [43, 44].

Earlier studies sought to sequence complete mitochondrial genomes, and detect variations in genomic structure, and summarize the patterns of gene arrangement. The researchers tended to focus on the diversity and unique structural features of the mitogenome, whilst paying little attention to the evolutionary implications of the mitogenomic structure. For example, the O_L_ disappeared in Scolecophidia species, but reappeared in Alethinophidia, which was not satisfactorily explained.

Recently, the number of assembled snake mitogenomes has increased rapidly, which provides an excellent opportunity to study the dynamic variation of mitogenomic features and their evolutionary implications. In the present study, we collected and sequenced complete snake mitogenomes from 65 species (including 3 new complete sequences in this study) to explore the occurrence of mitogenomic reorganizations. Significantly, we next investigated the evolutionary implications of mitogenome arrangements within snakes. Finally, we discuss potential evolutionary mechanisms responsible for mitogenome rearrangements and their effects on hotspot areas of rearrangement.

## Results

### Types of mitogenome arrangement

**Fig. 1:**
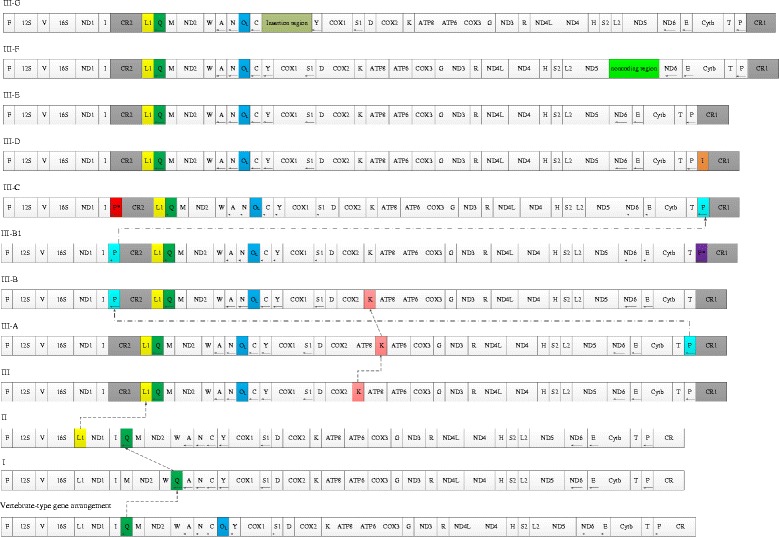
Comparison of mitochondrial gene organizations of snakes. The mitochondrial gene organization of snakes is illustrated as I, II, III, III-A, B, B1, C, D, E, F, G. Circular mitogenomes are represented linearly as bars and genes encoded by the H-strand and L-strand are shown, without and with the arrow, respectively. Genes, pseudogenes, control regions (CRs), non-coding regions, and light-strand replication origins are shown in boxes. The sizes of the boxes reflect the relative length of the genes and non-coding regions. Several genes relevant to discussions on gene rearrangements are highlighted in different color. Dotted arrows indicate the rearranged genes and the inferred evolutionary directions of the rearrangements

**Fig. 2:**
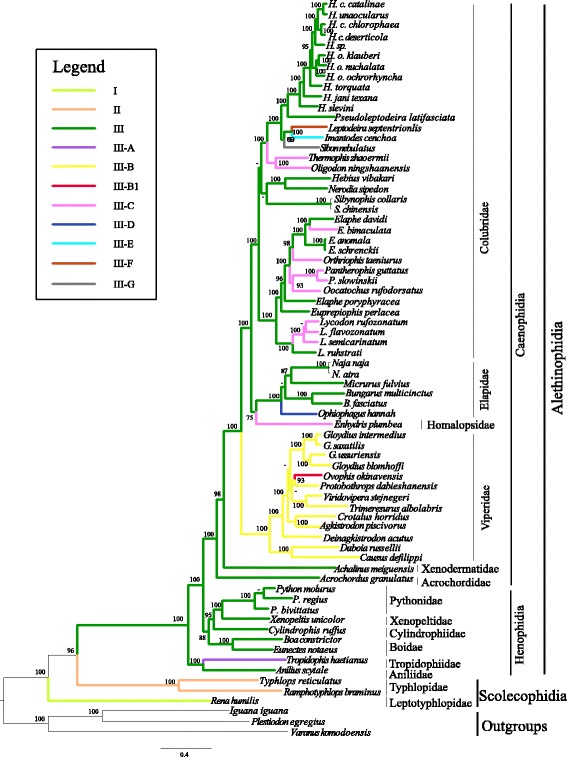
Maximum likelihood phylogenetic tree based on the combined data set of RNA and Protein-coding genes. Numbers above the lines or beside the nodes were bootstraps values. “-” indicates the values of maximum likelihood bootstrap proportions < 75. Types I to III-G correspond to those in Fig. 1

**Fig. 3:**
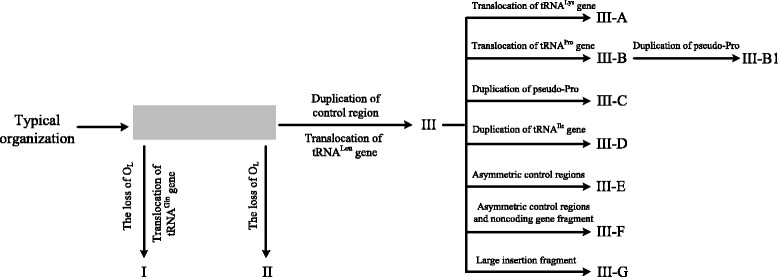
Putative evolutionary implications of mitochondrial genome rearrangement in snakes. The changes of mitogenomic rearrangements are shown. Types correspond to those in Fig. 2. The arrows denote the evolutionary directions of the rearrangements. The gray block represents the unknown/indeterminate ancestral condition of mitogenome arrangement

In the present study, the mitogenome size for *Lycodon ruhstrati*, *L. rufozinatum* and *L. flavozonatum* were found to range between 17,153-17,188 bp, respectively (Additional file 2: Table S1). The mitogenomes contained 2 rRNAs, 22 tRNAs, 13 protein-coding genes (PCGs), 2 control regions (CR1 and CR2), and a pseudo-pro (P*, which is absent in *L. ruhstrati*) (Additional file 2: Table S1). Disregarding the presence of P*, the composition and gene arrangements were the same as their sibling species, for example, *L. semicarinatus* (Additional file 2: Table S1, Fig. 1). In a comparative analysis of 65 mitogenome sequences in this study, eleven types of mitochondrial gene arrangements were found. According to their inferred evolutionary relationships in the phylogeny, the first three types were named Type I, Type II and Type III (Figs. 1, 2 and 3). A further eight types were named Type III-A, Type III-B, Type III-B1, Type III-C, Type III-D, Type III-E, Type III-F and Type III-G, according to their inferred evolutionary relationships with Type III (Figs. 1, 2 and 3). The gene organization pattern of each type is shown in Fig. 1 and discussed later in more detail.

### Phylogenetic analysis

The phylogenetic trees of snakes were reconstructed using Bayesian inferences and Maximum likelihood methods. Both phylogenetic methods provided identical and well-supported tree topologies (Fig. 2). The phylogenetic results indicate that Leptotyphlopidae is closest to the common ancestor rather than Typhlopidae. Thus, the Scolecophidia is a paraphyletic group. This result supports the non-monophyly of Scolecophidia as reported by other researchers [18, 45, 46]. Within Alethinophidia, three large clades are found, including a paraphyly of Henophidia and the monophyly of Caenophidia. Two families in Henophidia, Anillidae and Tropidophiidae, compose the basal clade to the other alethinophidians. The remaining alethinophidians are divided into two strongly supported clades, one containing the other four families (including Pythonidae, Xenopeltidae, Cylindrophiidae and Boidae) of Henophidia, the other represents the Caenophidia (advanced snakes).

The remaining species from the six families constituted a monophyletic lineage representing the Caenophidia. In this lineage, the Acrochordidae as sister to all other caenophidians, followed successively by Xenodermatidae, Viperidae, Homalopsidae, Elapidae and Colubridae. However, the resultant Bayesian tree showed that Homalopsidae was nested within Elapidae and Colubridae (Additional file 3: Figure S2). Thus, the topology of our phylogenetic tree is similar to previous studies [12, 18, 39, 46, 47] and in agreement with the results from nucelar gene analyses [45, 48].

### Types of mitogenomes and their phylogenetic distribution

Two mitogenome arrangement types, Type I and II, appeared in the Scolecophidians. Type I is distributed in the Leptotyphlopidae and located in the basal clade, and Type II only emerged in the Typhlopidae (Fig. 2). Type III was the dominant gene arrangement and extensively distributed in Alethinophidia (Fig. 2). Type III-A was only found in *Tropidophis haetianus* (Henophidia). Type III-B was the prevailing arrangement in Viperidae, and most species in Viperidae belong to this type except *O. okinavensis*, which possessed a new type (type III-B1). Type III-C is an arrestive arrangement type, widely distributed in the Colubridae and Homalopsidae. The phylogenetic tree indicated that type III-C occurred independently six times in Colubridae and Homalopsidae. Type III-D was identified in *O. hannah* (Elapidae). In addition, the remaining three Types, Type III-E, Type III-F and Type III-G, appeared in three Colubridae species (*I. cenchoa, L. septentrionalis, S. nebulatus*) respectively (Fig. 2).

### The evolutionary implications of mitogenome rearrangements

The evolutionary implications of the eleven mitogenome arrangements were inferred from their distribution situation in the phylogenetic tree of 65 species (Fig. 3). Type I originated from the unknown/indeterminate ancestral mitogenome arrangement, with two distinct features, the missing O_L_ and tRNA^Gln^ translocated from the *IQM* tRNA gene cluster to the *WANCY* cluster. Type II was also derived from the unknown/indeterminate ancestral mitogenome arrangement by losing O_L_ within the *WANCY* cluster (Figs. 1 and 3). Type III emerged in alethinophidians (Fig. 2), marked by two notable features, duplication of the control regions and translocation of the tRNA^Leu^ gene (Fig. 1). Just like Type I and II, the Type III was also derived from the unknown/indeterminate ancestral mitogenome arrangement pattern (Fig. 3). Obviously, the remaining types (Type III-A, -B, -B1, -C, −D, −E, -F, −G) were derived from Type III directly or indirectly, each with their own unique features (Fig. 3). On the basis of Type III, Type III-A translocated tRNA^Lys^ gene from 3′ proximity of *COX2* to 5′ proximity of *ATP6* gene. In Type III-B, the tRNA^Pro^ gene was translocated to the 5′ proximity of CR2. In *O. okinavensis*, the new type, Type III-B1 was derived from Type III-B, with a P* inserted into the 5′ proximity of CR1. When an additional P* was inserted into the 5′ proximity of CR2, Type III changes into the Type III-C. The Type III-D was derived from Type III, with an additional tRNA^Ile^ gene, which was duplicated from the 3 proximity of *ND1* and inserted to the 5 proximity of CR1. In Type III-E, the apparently prolonged asymmetric control regions (2878 bp in CR1, 4110 bp in CR2) were its typical characteristic. In Type III-F, a 342 bp noncoding gene fragment was found besides the asymmetry control regions. In Type III-G, a large insertion fragment (5702 bp) was inserted between the tRNA^Cys^ and tRNA^Tyr^ genes (Figs. 1 and 3).

## Discussion

### Eleven types of mitogenome arrangement within snakes

In this study, the mitogenomes of 65 snakes species from 14 families exhibited eleven gene arrangement patterns (Figs. 1, 2 and 3), which were more frequent than previously reported for snakes species. However, the distribution and frequency of each mitochondrial genome type is asymmetrically distributed across the phylogeny of snakes (Fig. 2). Type I and II only occurred in Scolecophidia and represented the potentially ancestral mitogenome arrangement pattern for snakes. Type III and its eight derivative types (Type III-A, III-B, III-B1, III-C, III-D, III-E, III-F, III-G) are distributed in the Alethinophidia (Fig. 2). Among them, some types have the preponderant distribution in a specific phylogenetic branch. For example, Type III was the common and prevailing arrangement in Alethinophidia, except in Viperidae where most arrangement patterns belonged to Type III-B.

In a previous study, six mitogenome arrangements were found in 14 snake species from11 families [18], moreover, in that study it seems that some families have fixed connections with specific mitogenome arrangement patterns. Undoubtedly, the expanded sample size in the present study provided more information on mitogenome diversity in snakes. Not only were more mitogenome arrangement patterns discovered but we also identified a more diverse phylogenetic distribution of patterns. In this study, five new mitogenome arrangements (Types III-A, III-D, III-E, III-F, III-G) were found in *T. haetianus* (Tropidophiidae), *O. hannah* (Elapidae), and in three Colubrid snakes, *I. cenchoa*, *L. septentrionalis*, *S. nebulatus*. Rare changes in mitogenome arrangement have attracted great interest because of their potential to provide homoplasy-free evidence of phylogenetic relationships [26]. Overall, in Caenophidia, almost every family (except those families containing just a single species) presented of multiple mitogenome arrangements, especially in Colubridae. Even within certain genera (e.g. *Lycodon* and *Elaphe*), there were multiple mitotypes (Type III and III-C) [16]. This phenomenon has also been identified in other groups, such as the presence of different mitotypes in a single Lizard genus (e.g. *Phrynocephalus*) [49]. However, many family-level taxa are represented by a single species, so it would be worthwhile to sequence more mitogenomes to explore mitogenomic diversity within these lineages.

### The dynamic evolution of snake mitogenome structure

Based on our phylogenetic reconstruction based on the mitogenomes of 65 species and the distribution of the eleven mitogenome arrangements across this topology (Figs. 1, 2 and 3), the evolutionary implications of mitogenome structure was inferred. Scolecophidia were the basal branches of all Alethinophidia snakes, and its mitogenome arrangement contains two types (Type I and II) (Fig. 2). Based on the phylogenetic results (Fig. 2), we surmise that Type I and II might be independently derived from some unknown ancestral mitogenome organization (Fig. 3). The most distinctive structural feature shared by Type I and II, is the missing O_L,_ which may have been derived independently from earlier snake mitogenomes.

Type III was the dominant type in Alethinophidia, which had two notable features, duplication of the control region and translocation of tRNA^Leu^ gene (Fig. 1). Notably, the mitochondrial genomes of Alethinophidia contain a stable O_L_ structure, which also has been found in lizard taxa [42] (Fig. 1, Additional file 4: Figure S3). It suggests that Type III may also be derived from the unknown ancestral mitogenomes by a tandem duplication of the control region and translocation of the tRNA^Leu^ gene (Figs. 1 and 3). The other eight types were derived directly or indirectly from Type III by translocation, duplication or insertion of specific genes (Fig. 3).

### Gene rearrangement hotspot I——WAN-O_L_-CY genes cluster

The WAN-O_L_-CY region, the cluster of five mitochondrial tRNA genes and the O_L_ among them (*tRNA*^*Trp*^, *tRNA*^*Ala*^, *tRNA*^*Asn*^, O_L_, *tRNA*^*Cys*^, *tRNA*^*Tyr*^, the typical order), has been revealed as a hotspot for gene order rearrangements by TDRL [26, 33]. These rearrangements involved translocations and insertions, which have been found in many vertebrate groups. For example, ACW-O_L_-NY, A-O_L_-CWNY and NCYWA-O_L_ were found in marsupials [26, 50], WNCYA-O_L_ and WA-O_L_-YNC in salamanders [33], WANYC (without O_L_) in Sloane’s viperfish (*Chaulioudus sloani*), and A-O_L_-WANCY in the dune gecko (*Stenodactylus petrii*) [6]. Insertion of a tRNA gene was found in the blind snake (*L. dulcis*), the tRNA^Gln^ was translocated from the IQM region (the gene cluster exists in typical vertebrate mitochondrial genomes including Typhlopidae, comprising tRNA genes, tRNA^Ile^, tRNA^Gln^ and tRNA^Met^) to the WANCY cluster, giving rise to a unique WQANCY gene order [42]. Length heteroplasmy has been reported in the WAN-O_L_-CY cluster. For example, a 66 bp element (pseudo-tRNA^Cys^) was inserted into O_L_ in the Actinopterygiian fish (*Pagellus bogaraveo*), which was explained by an independent translocation event through intra-mitochondrial recombination [51]. The pseudo-tRNA^Asn^ was also found in four caecilian amphibians (*Siphonops annulatus*, *S. paulensis*, *S. hardyi* and *Luetkenotyphlus brasiliensis*), but the presence of pseudo-tRNA^Asn^ was predicted by the TDRL model [26]. An extreme case was found in the clouded snake (*S. nebulatus*) where a large (5702 bp) insertion region with tandem repeats was detected between the tRNA^Cys^ and tRNA^Tyr^ genes and might have resulted from slipped-strand mispairing during mitogenome replication [43, 44, 52, 53].

Many previous studies have indicated that the O_L_ was possibly involved in the processes of the mitogenome molecule, such as gene rearrangements [26, 54], mutation gradients [55, 56] and nucleotide asymmetric compositional bias [57, 58]. For example, amphibians with unstable O_L_ were much more likely to have undergone gene rearrangements [59]. However, the absence of functional O_L_ has been reported in fishes, birds, crocodilians, lampreys, and some groups of lizards and snakes [42, 54, 60–62]. Previous studies have reported that the O_L_-like structure can act as the O_L_ when the regular O_L_ is lost (e.g. in Lepidosauria and *Symphurus*) [60, 63]. In this study, the O_L_ was lost in Scolecophidias, but found in all Alethinophidia species, which is in agreement with the results from Yan et al. [18]. However, the O_L_-like structure has not been detected in Scolecophidias, which differs from above studies [60, 63]. Previous molecular phylogenetic studies suggested a sister relationship between snakes and Lizards; the O_L_ was also found in all the lizard taxa [42]. Therefore, it may be inferred that the O_L_ is a plesiomorphy which persisted in Alethinophidia. In this study, based on the analysis of the O_L_ sequences of 65 snakes and 4 Lizards, we found that the O_L_ of Alethinophidia retains high sequence similarity to that of Lizards (Additional file 4: Figure S3). The loss of O_L_ both occurred in Typhlopidae and Leptotyphlopidae. In view that Scolecophidia is a paraphyletic group, loss of O_L_ should be two independent processes in the Scolecophidia lineages (Fig. 3).

### Gene rearrangement hotspot II——duplicate control regions and flanking tRNA genes

Duplications of control regions are often observed in various groups of vertebrates, such as fishes [14, 64], amphibians [3, 5], reptiles [17, 18, 41] and birds [7, 20, 65, 66]. In most cases, the copied CRs are highly similar to each other [5, 41, 65], and were usually interpreted as concerted evolution [16, 65, 67]. Generally, it was believed that concerted evolution was maintained by the tandem duplication and/or gene conversion via general recombination [41]. In the vast majority of snake mitogenomes, sequences of the control regions were almost identical to each other within each individual (orthologs), but very divergent in different individuals (paralogs), indicating that the duplicate control regions evolved concertedly. Dong et al. [17] proposed that such mechanisms operate on snake mitogenomes, the flanking tRNA genes may be copied together with the CR sequence to be pasted in other homologous regions of the mitogenome. In 2008, Kurabayashi et al. surveyed mantellid frogs and summarized that the control region was the hotspot of recombination and general recombination had a potential to cause gene rearrangement in upstream regions of multiple CRs as the results of gene conversion [31]. In the present study, amongst the 11 types of gene rearrangements, there are 7 types in which gene rearrangements occurred in the control regions and flanking segments, involving duplication of control regions and tRNA genes, translocation of tRNA genes, the presence of pseudogenes and asymmetry of the control regions (Fig. 1). Therefore, as for snake mitogenomes, the control regions and their adjacent segments were the hotspot for rearrangements, the maintenance mechanism of duplicate control regions is the source of mitogenome structural diversity.

The asymmetry of the duplicate control regions has been found in many species, often involving extensive tandem repeats or truncated genes [20]. In this study, asymmetric control regions were found in two colubrid species, *I. cenchoa* and *L. septentrionalis*. Considerable length variations exist in duplicate control regions, which are composed of hundreds of random repeats [44]. It was believed that tandem repeats may have resulted from slipped-strand mispairing during mitogenome replication [53, 68, 69], the changes of unit size and copy number can result in large size variations in control region sequences in birds and mammals [70–72]. Therefore, we think that asymmetry of duplicate control regions in snake mitogenomes can also be attributed to the presence of tandem repeats; at the same time it is also the source of gene rearrangement diversity.

In the present study, the pseudo-tRNA^Pro^ gene (P*), located at the 5′ proximity of CR2, was distributed in 6 independent lineages of the family Colubridae and Homalopsidae (Type III-C, Fig. 2). This result did not support previous studies, which reported that the P* was an exclusive feature of the Colubridae and Homalopsidae (Yan et al. [18], Additional file 1: Figure S1F). Combined with the phylogenetic analysis, we can see that the P* appeared seven times independently and successively (Figs. 2 and 3). It is worth noting that P* was polytopic and evolved independently in the evolutionary history of snakes. In Type III-B1 (*O. okinavensis*), P* located at the upstream of the CR1 and tRNA^Pro^ gene was translocated to the 5′ proximity of CR2 [17], it might result from gene conversion via general recombination. It is generally believed that the pseudogene remnants predicted by the TDRL model were uncommon in mitogenomes [33, 73, 74], for they were lost rapidly under strong selective pressure to constrain mitogenome size and gene number [26, 75]. In snake mitogenomes, the presence of pseudogenes may occur in two scenarios: first, the functional tRNA^Pro^ gene was duplicated with its associated CR, and then a portion of the tRNA^Pro^ gene was randomly deleted. Second, the tRNA^Pro^ gene was copied partially and pasted in the other homologous region of the mitogenome together with the CR sequence. In this study, the additional copy of the tRNA^Ile^ gene that was found in *O. hannah* might belong to the latter presupposition (Type III-D, Fig. 1), for it located in upstream of CR1.

## Conclusions

In the current study, the complete mitogenome sequences and structures of 65 snake species from 14 families were analyzed to examine their structural patterns, organization and evolution. Eleven types of mitochondrial gene arrangement pattern in total were found, which showed a trend of diversification of mitochondrial genome order rearrangements in snakes, especially in Alethinophidia. The snake mitogenome rearrangements mainly involved three processes, gene loss, translocation and duplication. Within Scolecophidia, the O_L_ was lost repeatedly in Typhlopidae and Leptotyphlopidae, but persisted as a plesiomorphy in Alethinophidia. Independently and stochastically, the presence of duplicate P* was a polytopic event in the mitogenome and emerged in seven different lineages of unequal size in three families. The *WANCY* tRNA gene cluster and the control regions and their adjacent segments were hotspots of mitogenome rearrangement. The maintenance mechanism of duplicate control regions of the mitogenome may be the source of its structural diversity.

## Methods

### Specimens used

In this study, the tissue samples of three species (*Lycodon ruhstrati*, *L. rufozonatum* and *L*. *flavozonatum*) were collected in the Dabie and Huangshan mountains between 2012 and 2014 (Additional file 5: Table S2). The above samples were preserved in 100% ethanol, then stored at − 20 °C after being transported to the laboratory until used for DNA extraction.

### DNA extraction, PCR amplification, sequencing

Tissues were washed with double-distilled water before DNA extraction, then total genomic DNA was extracted using the standard phenol/chloroform method [76]. Total DNA was examined on 1.0% EB-agarose gels and stored at −20°C. Nineteen pairs of universal primers were designed to amplify and sequence the complete mitochondrial genome of *L*. *ruhstrati*, *L. rufozonatum* and *L*. *flavozonatum* (Additional file 6: Table S3).

PCR reactions were carried out in 50 μl reaction volumes containing 1 μl (50-80 ng) template DNA, 25 μl 2 × Easy*Taq* PCR SuperMix polymerase (TransGen Biotech, containing 1.25 U Ex *Taq*, 0.4 mM dNTP, 4 mM Mg^2+^), 1 μl of each 10 mM primer, and sterile double-distilled water to final volume. PCRs were performed in a PCR-Cycler (TC-96/G/H(b)C) and amplification conditions were as follows: initial denaturation for 5 min at 95 °C, followed by 32 cycles of denaturation for 30s at 95 °C, 51-54 °C for 40s (annealing), and 72 °C for 80-100 s (extension), and a final extension step of 10 min at 72 °C. PCR products were examined by EB-agarose gel electrophoresis to validate amplification efficiency. After purification, all the products were sent to Sangon Biotech Company (Shanghai, China) for sequencing from both directions using the primers in the PCR amplification.

### Sequence assembly and collection

Contiguous fragments were assembled to create complete mitochondrial genomes in Seqman II (DNAStar, Madison, WI, USA) and checked by visual inspection to ensure the accuracy of variable sites identified by the program [77]. The harvested mitogenome sequences have been deposited in the GenBank, and the accession numbers of *L. ruhstrati*, *L. rufozinatum* and *L. flavozonatum* were KJ179951, KJ179950 and KR911720, respectively. The mitochondrial genomes of other 62 species from 14 families were downloaded from Genbank (Additional file 5: Table S2, Classification system followed Pyron et al. [48]).

### Data analysis

Genes encoded in the determined mitogenome sequences were identified by comparison with corresponding gene sequences from other snakes and subsequent manual inspection of gene structure [18, 41]. In addition, the software Getmitogenome was used to excise nucleotide sequences of encoded genes, as well as amino acid sequences of 13 protein genes, which were added to a pre-existing alignment dataset [6]. The base composition, codon usage, and open-reading frames (ORF) were analyzed using program MEGA 5.0 [78]. The overlapping regions and intergenic spacers were counted manually [79]. The tRNA genes were identified using the software package tRNAscan-SE 2.0 (http://lowelab.ucsc.edu/tRNAscan-SE/) by eye, based on vertebrate mitochondrial anti-codon sequences and their secondary structure. In addition, the DOGMA annotation software was used to check annotated genes [80]. This procedure helped us to evaluate gene boundaries more carefully and to identify possible pseudo-genes [6]. The genome arrangements with same components and order were classified as identical type. All mitogenome types were visualized by linearized organization, and drawn by Microsoft Visio.

### Phylogeny reconstruction

Twelve protein-coding, 22 tRNA, and two rRNA gene sequences were separately aligned using Clustal X 1.8 software with default settings followed by manual adjustment [39, 46, 81], except *ND6* gene and control regions because of their heterogeneous base composition and poor phylogenetic performance [57, 82]. For each dataset, best fit combinations of partitioning schemes and nucleotide substitution models were determined with PartitionFinder version 2.1.1 [83], using the “greedy” algorithm [84] and the Bayesian information criterion (BIC), with the branch lengths of alternative partitions linked and with the software set to evaluate specific substitution model sets for either RAxML or MrBayes independently. Best-fit substitution models and partitioning schemes selected in each case are given in Additional file 7: Table S4.

Phylogenetic analyses were carried out by Bayesian and Maximum Likelihood (ML) methods, using *Iguana iguana, Plestiondon egregius* and *Varanus komodoensis* as outgroups [18]. The Bayesian analyses were implemented with MrBayes version3.1.2 [85]. MrBayes analyses simultaneously initiate two Markov Chain Monte Carlo (MCMC) model runs to provide additional confirmation of convergence of posterior probability distributions. Analyses were run for 10,000,000 generations. Chains were sampled every 1000 generations. When the average standard deviation of split frequencies reached a value less than 0.01, the first 1000 trees were discarded as “burn-in” and the remaining trees were used to calculate Bayesian posterior probabilities [86, 87]. The maximum likelihood (ML) analysis was performed using an algorithm in the RAxML software [88, 89] under the GTR + I + G model, and the robustness of the phylogenetic results were tested through bootstrap analysis with 1000 replications [90].

Combining the definite phylogenetic relationships of different species, with the distributed information of each mitogenome type in the phylogenetic lineage of snakes, we infer the evolutionary implications of the mitogenome structure and represent it by schematic diagram.

## Additional files

Additional file 1: **Figure S1.** Gene organization of control regions and *WANCY* cluster in snake mitochondrial genomes. Circular mitogenomes are linearly depicted as an open bar divided into individual genes. Only relevant genes are shown, and in a way that does not reflect actual gene lengths. B, C, D, E came from Kumazawa et al. [16]; A, F from Yan et al. [18]; G from Chen and Zhao [19]. The H- and L- strand encoded genes are denoted above and below each gene box. Transfer RNAs are indicated by their single-letter abbreviations. Abbreviations: 12S, 16S, and P* stand for 12S rRNA, 16S rRNA, and a pseudogene for tRNAPro gene, respectively. Taxa for which have been reported to date are listed in Additional file 5: Table S2. (PDF 612 kb)

Additional file 2: **Table S1.** Features of the mitogenomes of three *Lycodon* species. (DOCX 22 kb)

Additional file 3: **Figure S2.** Bayesian phylogenetic inference tree based on the combined data set of RNA genes and Protein-coding genes. The numbers above the branches indicate the posterior probability. (PDF 256 kb)

Additional file 4: **Figure S3.** Homology analysis of O_L_. The sequences of OL of Alethinophidians and four saurians are aligned. (PDF 574 kb)

Additional file 5: **Table S2.** List of taxa used in this study. *: the species were used in Yan et al. [18]. #: the species were used in Chen and Zhao. [19]. (DOC 132 kb)

Additional file 6: **Table S3.** Primers sequences used in this study. (DOC 66 kb)

Additional file 7: **Table S4.** Best-fit models and partitioning schemes selected by PartitionFinder for the dataset analyzed. (DOCX 22 kb)

## Abbreviations

12S: 12S ribosomal RNA
16S: 16S ribosomal RNA
ATP6: ATPase subunit 6
ATP8: ATPase subunit 8
COX1-3: Cytochrome c oxidase subunits 1-3
CR: Control region; Transfer RNA genes are depicted with the corresponding single-letter amino acid
Cyt *b*: Cytochrome b
IQM: tRNA^Ile^, tRNA^Gln^, tRNA^Met^
L1: tRNA^Leu (UUR)^
L2: tRNA^Leu (CUN)^
ND1-6: NADH dehydrogenase subunits 1-6
ND4L: NADH dehydrogenase subunits 4 L
O_L_: The putative L-strand replication origin
*P**: A pseudogene of tRNA^Pro^ gene
S1: tRNA^Ser (UCN)^
S2: tRNA^Ser (AGY)^
TDNL: Non-Random Loss
TDRL: Tandem Duplication and Random Loss
WANCY: tRNA^Trp^, tRNA^Ala^, tRNA^Asn^, tRNA^Cys^ and tRNA^Tyr^
