# Supplementary material for: Multiple independent structural dynamic events in the evolution of snake mitochondrial genomes
Source: BMC Genomics. 2018 May 10;19:354. doi: 10.1186/s12864-018-4717-7 (PMC5946542; doi:10.1186/s12864-018-4717-7)
Supplement: Supplementary file 1 — Figure S1. Gene organization of control regions and WANCY cluster in snake mitochondrial genomes. Circular mitogenomes are linearly depicted as an open bar divided into individual genes. Only relevant genes are shown, and in a way that does not reflect actual gene lengths. B, C, D, E came from Kumazawa et al. [16]; A, F from Yan et al. [18]; G from Chen and Zhao [19]. The H- and L- strand encoded genes are denoted above and below each gene box. Transfer RNAs are indicated by their single-letter abbreviations. Abbreviations: 12S, 16S, and P* stand for 12S rRNA, 16S rRNA, and a pseudogene for tRNAPro gene, respectively. Taxa for which have been reported to date are listed in Additional file 5: Table S2. (PDF 612 kb) [file 12864_2018_4717_MOESM1_ESM.pdf]

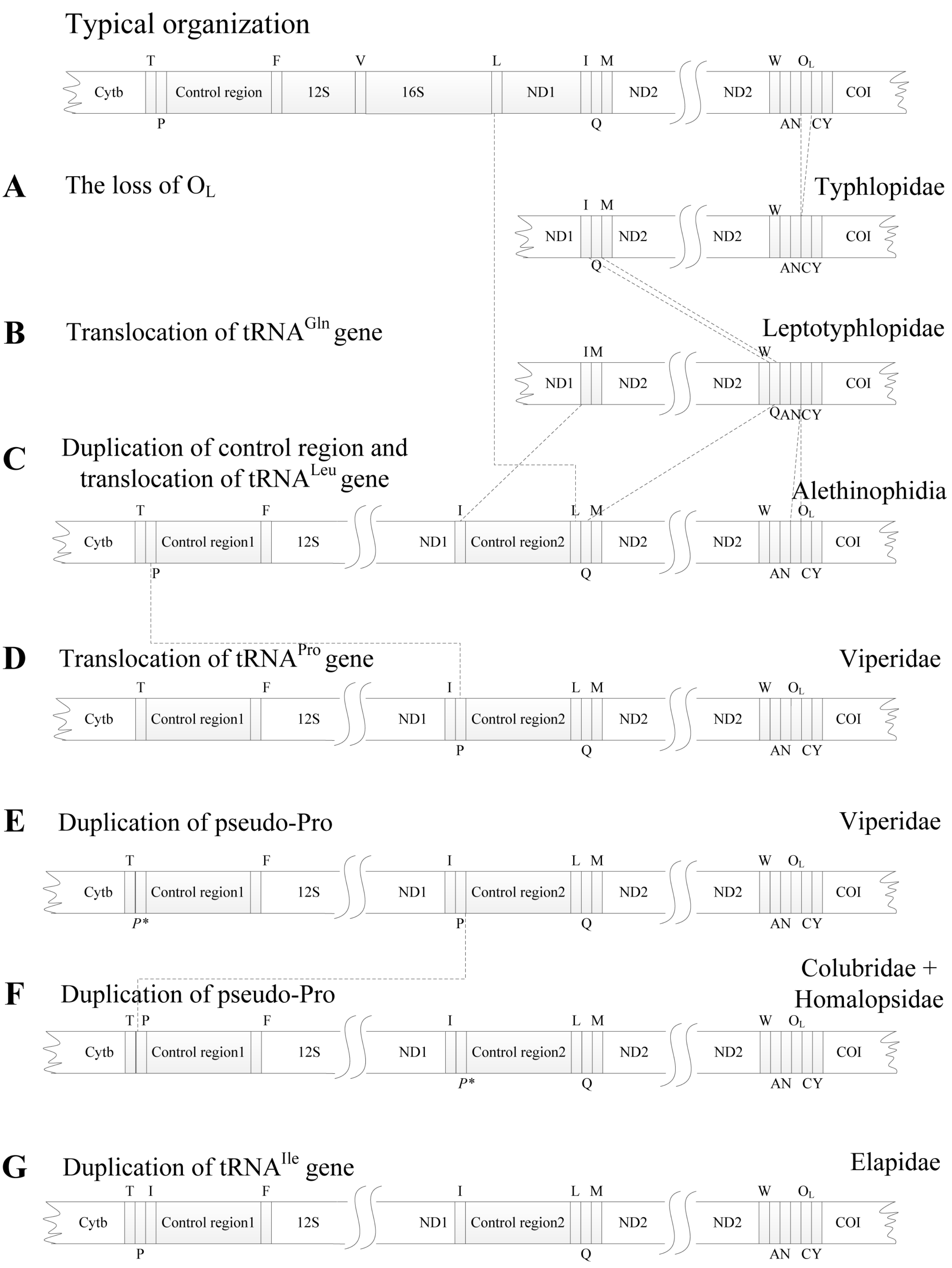

Typical organization
T F V L I M W O_L
Cytb
Control region
12S
16S
ND1
ND2
ND2
COI
P Q AN CY
A
The loss of O_L
Typhlopidae
I M W
ND1 ND2 ND2 COI
Q ANCY
B
Translocation of tRNA^Gln gene
Leptotyphlopidae
IM W
ND1 ND2 ND2 COI
QANCY
C
Duplication of control region and
translocation of tRNA^Leu gene
Alethinophidia
T F I L M W O_L
Cytb Control region1 12S ND1 Control region2 ND2 ND2 COI
P Q AN CY
D
Translocation of tRNA^Pro gene
Viperidae
T F I L M W O_L
Cytb Control region1 12S ND1 Control region2 ND2 ND2 COI
P Q AN CY
E
Duplication of pseudo-Pro
Viperidae
T F I L M W O_L
Cytb Control region1 12S ND1 Control region2 ND2 ND2 COI
P* P Q AN CY
F
Duplication of pseudo-Pro
Colubridae +
Homalopsidae
T P F I L M W O_L
Cytb Control region1 12S ND1 Control region2 ND2 ND2 COI
P* Q AN CY
G
Duplication of tRNA^Ile gene
Elapidae
T I F I L M W O_L
Cytb Control region1 12S ND1 Control region2 ND2 ND2 COI
P Q AN CY
