# Supplementary material for: Multiple independent structural dynamic events in the evolution of snake mitochondrial genomes
Source: BMC Genomics. 2018 May 10;19:354. doi: 10.1186/s12864-018-4717-7 (PMC5946542; doi:10.1186/s12864-018-4717-7)
Supplement: Supplementary file 2 — Table S1. Features of the mitogenomes of three Lycodon species. (DOCX 22 kb) [file 12864_2018_4717_MOESM2_ESM.docx]

**Table S1 Features of the mitogenomes of three *Lycodon* species**

| Gene/strand | Position | | |  | Length | | |  | Start/stop codon | | |
| --- | --- | --- | --- | --- | --- | --- | --- | --- | --- | --- | --- |
|  | *L. ruh* | *L.ruf* | *L.fla* |  | *L. ruh* | *L.ruf* | *L.fla* |  | *L. ruh* | *L.ruf* | *L.fla* |
| tRNA^Phe/^H | 1-64 | 1-66 | 1-66 |  | 64 | 66 | 66 |  |  |  |  |
| 12S rRNA/H | 65-991 | 65-999 | 65-997 |  | 927 | 935 | 933 |  |  |  |  |
| tRNA^Val^/H | 992-1057 | 1000-1063 | 998-1061 |  | 66 | 64 | 64 |  |  |  |  |
| 16S rRNA /H | 1058-2526 | 1064-2538 | 1062-2537 |  | 1469 | 1475 | 1476 |  |  |  |  |
| ND1/H | 2527-3490 | 2539-3502 | 2538-3501 |  | 964 | 964 | 964 |  | ATA/T | ATA/T | ATA/T |
| tRNA^Ile^/H | 3491-3556 | 3503-3568 | 3502-3566 |  | 66 | 66 | 65 |  |  |  |  |
| Pseudo-Pro/L | - | 3565-3600 | 3567-3597 |  | - | 36 | 31 |  |  |  |  |
| Control region2/H | 3556-4575 | 3601-4619 | 3598-4618 |  | 1020 | 1019 | 1021 |  |  |  |  |
| tRNA^Leu(UUR)^/H | 4576-4649 | 4620-4692 | 4619-4691 |  | 74 | 73 | 73 |  |  |  |  |
| tRNA^Gln^/L | 4651-4721 | 4694-4764 | 4693-4763 |  | 71 | 71 | 71 |  |  |  |  |
| tRNA^Met^/H | 4723-4785 | 4766-4827 | 4765-4826 |  | 63 | 62 | 62 |  |  |  |  |
| ND2/H | 4786-5815 | 4828-5857 | 4827-5856 |  | 1030 | 1030 | 1030 |  | ATT/T | ATT/T | ATT/T |
| tRNA^Trp^/H | 5816-5880 | 5858-5922 | 5857-5922 |  | 65 | 65 | 66 |  |  |  |  |
| tRNA^Ala^/L | 5883-5947 | 5924-5988 | 5924-5988 |  | 65 | 65 | 65 |  |  |  |  |
| tRNA^Asn^/L | 5948-6019 | 5989-6060 | 5989-6060 |  | 72 | 72 | 72 |  |  |  |  |
| O_L_/L | 6022-6055 | 6063-6097 | 6061-6095 |  | 34 | 35 | 35 |  |  |  |  |
| tRNA^Cys^/L | 6054-6114 | 6096-6156 | 6096-6156 |  | 61 | 61 | 61 |  |  |  |  |
| tRNA^Tyr^/L | 6114-6175 | 6156-6217 | 6156-6217 |  | 62 | 62 | 62 |  |  |  |  |
| COI/H | 6177-7722 | 6219-7764 | 6219-7764 |  | 1546 | 1546 | 1546 |  | GTG/T | GTG/T | GTG/T |
| tRNA^Ser(UCN)^/L | 7769-7834 | 7811-7876 | 7811-7876 |  | 66 | 66 | 66 |  |  |  |  |
| tRNA^Asp^/H | 7835-7897 | 7877-7939 | 7877-7939 |  | 63 | 63 | 63 |  |  |  |  |
| COII/H | 7898-8582 | 7940-8624 | 7940-8624 |  | 685 | 685 | 685 |  | ATG/T | ATG/T | ATG/T |
| tRNA^Lys^/H | 8583-8644 | 8625-8687 | 8625-8687 |  | 62 | 63 | 63 |  |  |  |  |
| ATP8/H | 8646-8804 | 8689-8847 | 8689-8847 |  | 159 | 159 | 159 |  | ATG/TAA | ATG/TAA | ATG/TAA |
| ATP6/H | 8795-9475 | 8838-9518 | 8838-9518 |  | 681 | 681 | 681 |  | ATG/TAA | ATG/TAA | ATG/TAA |
| COIII/H | 9475-10258 | 9518-10301 | 9518-10301 |  | 784 | 784 | 784 |  | ATG/T | ATG/T | ATG/T |
| tRNA^Gly^/H | 10259-10319 | 10302-10362 | 10302-10362 |  | 61 | 61 | 61 |  |  |  |  |
| ND3/H | 10320-10662 | 10363-10705 | 10363-10705 |  | 343 | 343 | 343 |  | ATT/T | ATT/T | ATT/T |
| tRNA^Arg^/H | 10663-10727 | 10706-10770 | 10706-10770 |  | 65 | 65 | 65 |  |  |  |  |
| ND4L/H | 10728-11017 | 10771-11060 | 10771-11061 |  | 290 | 290 | 290 |  | GTG/TA | ATG/TA | ATG/TA |
| ND4/H | 11018-12355 | 11061-12398 | 11061-12398 |  | 1338 | 1338 | 1338 |  | ATG/TAG | ATG/TAA | ATG/TAA |
| tRNA^His^/H | 12355-12420 | 12399-12464 | 12399-12464 |  | 66 | 66 | 66 |  |  |  |  |
| tRNA^Ser(AGY)^/H | 12421-12477 | 12465-12522 | 12465-12522 |  | 57 | 58 | 58 |  |  |  |  |
| tRNA^Leu(CUN)^/H | 12475-12545 | 12520-12590 | 12520-12590 |  | 71 | 71 | 71 |  |  |  |  |
| ND5/H | 12546-14318 | 12591-14363 | 12591-14363 |  | 1773 | 1773 | 1773 |  | ATG/AGG | ATG/AGG | ATG/AGG |
| ND6/L | 14310-14813 | 14355-14852 | 14355-14855 |  | 504 | 498 | 501 |  | ATG/AGG | ATG/AGA | ATG/AGA |
| tRNA^Glu^/L | 14823-14884 | 14862-14923 | 14865-14926 |  | 62 | 62 | 62 |  |  |  |  |
| Cytb/H | 14885-16001 | 14924-16040 | 14928-16044 |  | 1117 | 1117 | 1117 |  | ATG/T | ATG/T | ATG/T |
| tRNA^Thr^/H | 16002-16066 | 16041-16105 | 16045-16116 |  | 65 | 65 | 72 |  |  |  |  |
| tRNA^Pro^/L | 16067-16128 | 16106-16167 | 16090-16151 |  | 62 | 62 | 62 |  |  |  |  |
| Control region1/H | 16129-17153 | 16168-17188 | 16152-17172 |  | 1025 | 1021 | 1021 |  |  |  |  |
