# Supplementary figures and images for: Multiple independent structural dynamic events in the evolution of snake mitochondrial genomes
Source: BMC Genomics. 2018 May 10;19:354. doi: 10.1186/s12864-018-4717-7 (PMC5946542; doi:10.1186/s12864-018-4717-7)

Legend

I

II

III

III-A

III-B

III-B1

III-C

III-D

III-E

III-F

III-G

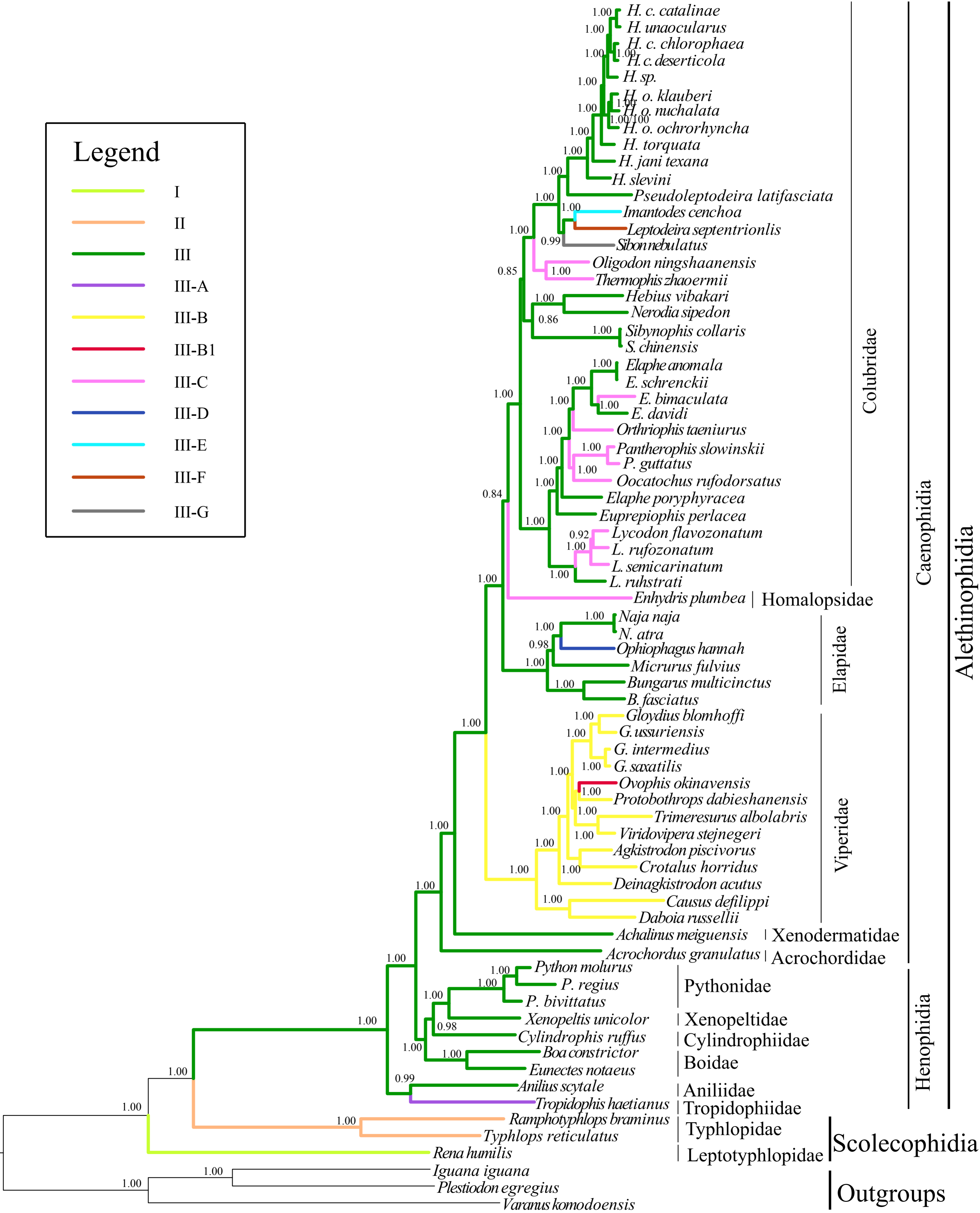

Supplement: Supplementary file 3 — Figure S2. Bayesian phylogenetic inference tree based on the combined data set of RNA genes and Protein-coding genes. The numbers above the branches indicate the posterior probability. (PDF 256 kb) [file 12864_2018_4717_MOESM3_ESM.pdf]
