# Supplementary material for: Multiple independent structural dynamic events in the evolution of snake mitochondrial genomes
Source: BMC Genomics. 2018 May 10;19:354. doi: 10.1186/s12864-018-4717-7 (PMC5946542; doi:10.1186/s12864-018-4717-7)
Supplement: Supplementary file 4 — Figure S3. Homology analysis of OL. The sequences of OL of Alethinophidians and four saurians are aligned. (PDF 574 kb) [file 12864_2018_4717_MOESM4_ESM.pdf]

```
L.rufozonatu...GC.TTCTCC.GTTTTT.AAACGTAGGG.....AAAAAA...CGG.A   34
L.semicarina...GC.TTCTCC.GTTTTT.GGACGAAAG......AAAAAA...CGG.A   33
L.flavozonat...GC.TTCTCC.GTTTTT.GAGCTGGGGG.....AAAAAA...CGG.A   34
L.ruhstrati ...GC.TTCTCC.GTTTTT.GGACGGGGGG.....AAAAA....CGG.A   33
H.torquata  ...GC.TTCTCC.GTTTTT.GCGAGCTG.......AAAAAAAA.CGG.A   34
H.o.ochrorhy...GC.TTCTCC.GTTTTT.GTAAGCTGAG.....AAAAAA...CGG.A   34
H.o.nuchalat...GC.TTCTCC.GTTTTT.GGGCAGGAG......AAAAAA...CGG.A   33
H.j.texana  ...GC.TTCTCC.GTTTTT.GTTAGGCCGG.....AAAAAA...CGG.A   34
H.c.chloroph...GC.TTCTCC.GTTTTT.GTTAGGCCTGG....AAAAAA...CGG.A   35
H.sp.       ...GC.TTCTCC.GTTTTT.GTTAGGCTGG.....AAAAAA...CGG.A   34
H.c.desertic...GC.TTCTCC.GTTTTT.GTTAGGGCTGG....AAAAAA...CGG.A   35
H.unaocularu...GC.TTCTCC.GTTTTT.GTTAGGCTGG.....AAAAAA...CGG.A   34
H.c.catalina....C.TTCTCC.GTTTTT.GTTAGGCTGG.....AAAAAA...CGG.A   33
H.slevini   ...GC.TTCTCC.GTTTTT.GTTAGCCTAGG....AAAAAA...CGG.A   35
H.o.klauberi...GC.TTCTCC.GTTTTT.GAGGGGGGGGG....AAAAA....CGG.A   34
T.zhaoermii ...GC.TTCTCC.GTTTTT.GGGCGGGCGGG....AAAAA....CGG.A   34
P.latifascia...GC.TTCTCC.GTTTTT.GGGCGGGGGG.....AAAAAA...CGG.A   34
O.ningshaane...GC.TTCTCC.GTTTTT.GAGCTGGGGG.....AAAAAA...CGG.A   34
H.v.ruthveni...GC.TTCTCC.GTTTTT.GGGCGGGGGG.....AAAAAA...CGG.A   34
E.anomala   ...GC.TTCTCC.GTTTTT.GAGCTGGGGG.....AAAAAA...CGG.A   34
E.schrenckii...GC.TTCTCC.GTTTTT.GAGCAGGGGG.....AAAAAA...CGG.A   34
O.rufodorsat...GC.TTCTCC.GTTTTT.GGGCGGGGGG.....AAAAAA...CGG.A   34
O.taeniurus ...GC.TTCTCC.GTTTTT.GGGCGGGGGG.....AAAAAA...CGG.A   34
E.perlacea  ...GC.TTCTCC.GTTTTT.GGGCGGGGGG.....AAAAAA...CGG.A   34
E.poryphyrac...GC.TTCTCC.GTTTTT.GGGCGGTGG......AAAAAA...CGG.A   33
S.collaris  ...GC.TTCTCC.GTTTTT.GGCCGGGAGAG....AAAAA....CGG.A   34
S.chinensis ...GC.TTCTCC.GTTTTT.GGCCGGGAGAG....AAAAA....CGG.A   34
P.slowinskii...GC.TTCTCC.GTTTTT.GTCACGGTATAG...AAAAA....CGG.A   35
P.g.guttatus...GC.TTCTCC.GTTTTT.GTCACGGTATAG...AAAAA....CGG.A   35
E.davidi    ...GC.TTCTCC.GTTTT..GTATGGGAG......AAAAAA...CGG.A   32
E.bimaculata...GC.TTCTCC.GTTTTT.AAAAGGGGGG.....AAAAAA...CGG.A   34
I.cenchoa   ...GC.TTCTCC.GTTTTTTGAACGGGAAAG....AAAAA....CGG.A   35
S.nebulatus ...GC.TTCTCC.GTTTTT.GAAACGGG.......AAAAAAAA.CGG.A   34
N.sipedon   ...GCTTTCTCC.GTTTTT.GGGCGGGGGG.....AAAAAA...CGG.A   35
L.s.polystic...GC.TTCTCC.GTTTTT.ATTAAAACGGGG...AAAAAA...CGG.A   36
B.fasciatus ...GCTTTCTCC.GTTTTT.ATAAAAAGG......AAAAAAAA.CGG.A   36
B.multicinct...GCTTTCTCC.GTTTT..GTGGGGGG.......AAAAAAA..CGG.A   33
N.atra      ...GCTTTCTCC.GTTTTT.GTAACGGG.......AAAAAAAAACGG.A   36
N.naja      ...GCTTTCTCC.GTTTTT.GTAACGGG.......AAAAAAAAACGG.A   36
M.fulvius   ...GCTTTCTCC.GTTTTT.ATTAACGATGGG...AAAAAA...CGG.A   37
O.hannah    ...GCTTTCTCC.GTTTTT.ATAACGGAAGG....AAAAAA...CGG.A   36
E.plumbea   ...GC.TTCTCC.GTTTT..AAGTCACGGC.....AAAAAAAAACGG.A   36
P.dabieshane...CC.TTCTCC.GTTTTT.GGGCGGGGGG.....AAAAAA...CGG.A   34
O.okinavensi...CC.TTCTCC.GTTTTT.GGGGGGAAG......AAAAAA...CGG.A   33
T.albolabris...CC.TTCTCC.GTTTTT.GGGGGGGGG......AAAAAA...CGG.A   33
G.ussuriensi..GCC.TTCTCC.GTTTTTTGGAGGGAAG......AAAAAAAA.CGG.A   37
G.b.brevicau..GTC.TTCTCC.GTTTTT.GGGGGGAAG......AAAAAAA..CGG.A   35
V.s.stejnege...CC.TTCTCC.GTTTTT.GGGGGGGGGG.....AAAAAA...CGG.A   34
G.intermediu..GCC.TTCTCC.GTTTTTTGGGGGGGAGG.....AAAAAAA..CGG.A   37
G.saxatilis ..GCC.TTCTCC.GTTTTT.GGGGGGAAG......AAAAAAA..CGG.A   35
A.piscivorus...CC.TTCTCC.GTTTTT.GAGCGGAG.......AAAAAAA..CGG.A   33
C.horridus  ...GC.TTCTCC.GTTTTT.GGGCGCCGGG.....AAAAAA...CGG.A   34
D.acutus    ...CC.TTCTCC.GTTTTT.GGGCGGGGGG.....AAAAAA...CGG.A   34
C.defilippi ...CC.TTCTCC.GTTTTT.GTAGGGGGGGG....AAAAAA...CGG.A   35
D.russellii ....C.TTCTCC.GTTTTT.GGCCGGGGAG.....AAAAAA...CGG.A   33
A.granulatus..TAC.TTCTCC.GTTTTT.AAACAGAAGT.....AAAAA....CGG.A   34
P.regius    ....C.TTCTCC.GTTTT...AACAAAG.......AAAAAA...CGG.A   29
P.m.molurus ....C.TTCTCC.GTTTT...AACAAG........AAAAAAA..CGG.A   29
P.bivittatus....C.TTCTCC.GTTTTT..AACAAAAG......AAAAAAA..CGG.A   32
X.unicolor  ....C.TTCTCC.GTTTT...AACGT.........AAAAAAAA.CGG.A   29
C.ruffus    ....C.TTCTCC.GTTTT....ACGGG........AAAAAA...CGG.A   27
E.notaeus   ....C.TTCTCC.GTTTT...AACGGAG.......AAAAAA...CGG.A   29
B.constricto....C.TTCTCC.GTTTT...GCCAGAG.......AAAAAA...CGG.A   29
A.meiguensis....CTTTCTCC.GTTTTT.CCTAGGGGAAAAAAGAAAAA....CGG.A   38
T.haetianus ..ATC.TTCTCC.GTTTTT.AAACGGGAAG.....AAAAA....CGG.A   34
A.scytale   ....C.TTCTCC.GTTTT..AAAGG..........AAAAAAA..CGG.A   28
A.fragilis  .TCTC.TTCTCCCGTT....AGT............AAAAAA...CGGGA   28
I.iguana    ....C.TTCTCCCGTT....GATAAG.........AAA......CGGGA   25
P.egregius  ..CGC.TTCTTCTGTT....AGA.....................AGA.A   20
V.komodoensi....C.TTCTCCCGTTTTT.AAAAGGGAG......AAAA.....CGGGA   32
Consensus      gc ttctcc gttttt gggcggggg      aaaaaa   cgg a
```
