# Supplementary material for: Multiple independent structural dynamic events in the evolution of snake mitochondrial genomes
Source: BMC Genomics. 2018 May 10;19:354. doi: 10.1186/s12864-018-4717-7 (PMC5946542; doi:10.1186/s12864-018-4717-7)
Supplement: Supplementary file 5 — Table S2. List of taxa used in this study. *: the species were used in Yan et al. [18]. #: the species were used in Chen and Zhao. [19]. (DOC 132 kb) [file 12864_2018_4717_MOESM5_ESM.doc]

**Table S2 List of taxa used in this study**

| **Family** | **Species** | **Genbank Accession No.** | **Reference** | **Type** |
| --- | --- | --- | --- | --- |
| Ingroup |  |  |  |  |
| **Scolecophidia** |  |  |  |  |
| Leptotyphlopidae | *Rena humilis^#^* | NC_005961 | Castoe et al., 2008 | I |
| Typhlopidae | *Ramphotyphlops braminus^*,#^* | NC_010196 | Yan et al., 2008 | II |
|  | *Typhlops reticulatus^#^* | NC_010971 | Kumazawa and Endo, 2004 | II |
| **Alethinophidia** |  |  |  |  |
| **Henophidia** |  |  |  |  |
| Aniliidae | *Anilius scytale* | NC_014343 | Castoe et al., 2009 | III |
| Tropidophiidae | *Tropidophis haetianus* | NC_012573 | Castoe et al., 2009 | III-A |
| Boidae | *Boa constrictor^*,#^* | NC_007398 | Dong and Kumazawa, 2005 | III |
|  | *Eunectes notaeus* | AM236347 | Douglas et al., 2006 | III |
| Cylindrophiidae | *Cylindrophis ruffus^*,#^* | NC_007401 | Dong and Kumazawa, 2005 | III |
| Xenopeltidae | *Xenopeltis unicolor^*,#^* | NC_007402 | Dong and Kumazawa, 2005 | III |
| Pythonidae | *Python bivittatus* | KF293729 | Liu et al., 2013, | III |
|  | *P. molurus* | NC_015812 | Dubey et al., 2011 | III |
|  | *P. regius^*,#^* | NC_007399 | Dong and Kumazawa, 2005 | III |
| **Caenophidia** |  |  |  |  |
| Acrochordidae | *Acrochordus granulatus^*,#^* | NC_007400 | Dong and Kumazawa, 2005 | III |
| Xenodermatidae | *Achalinus meiguensis^#^* | NC_011576 | Wang et al., 2009 | III |
| Viperidae | *Daboia russellii^#^* | NC_011391 | Chen and Fu, 2009 | III-B |
|  | *Causus defilippi* | NC_013479 | Castoe et al., 2009 | III-B |
|  | *Deinagkistrodon acutus^*,#^* | NC_010223 | Yan et al., 2008 | III-B |
|  | *Trimeresurus albolabris* | NC_022820 | Song et al., 2013 | III-B |
|  | *Trimeresurus stejnegeri* | NC_012146 | Lin et al., 2009 | III-B |
|  | *Ovophis okinavensis^*,#^* | NC_007397 | Dong and Kumazawa, 2005 | III-B1 |
|  | *Protobothrops dabieshanensis* | NC_022473 | Huang et al., 2014 | III-B |
|  | *Crotalus horridus* | NC_014400 | Hall et al., 2013 | III-B |
|  | *Gloydius saxatilis* | NC_025666 | Xu and Zhao, 2014, | III-B |
|  | *G. intermedius* | NC_025560 | Xu and Zhao, 2014, | III-B |
|  | *G. blomhoffi ^#^* | NC_011390 | Chen and Fu, 2009 | III-B |
|  | *G. ussuriensis* | NC_026553 | Xu and Zhao, 2015 | III-B |
|  | *Agkistrodon piscivorus^*,#^* | NC_009768 | Jiang et al., 2007 | III-B |
| Homalopsidae | *Enhydris plumbea^*,#^* | NC_010200 | Yan et al., 2008 | III-C |
| Elapidae | *Micrurus fulvius* | NC_013481 | Castoe et al., 2009 | III |
|  | *Ophiophagus hannah^#^* | EU921899 | Chen and Lai, 2010 | III-D |
|  | *Naja naja^*,#^* | NC_010225 | Yan et al., 2008 | III |
|  | *N. atra* | EU913475 | Chen and Fu, 2008, | III |
|  | *Bungarus multicinctus^#^* | NC_011392 | Chen et al., 2009 | III |
|  | *B. fasciatus^#^* | NC_011393 | Chen et al., 2009 | III |
| Colubridae | *Sibynophis collaris* | NC_016424 | Jang and Hwang, 2011 | III |
|  | *S. chinensis* | KF360246 | Oh et al., 2013 | III |
|  | *Oligodon ningshaanensis* | NC_026083 | Wang and Zhu, 2015, | III-C |
|  | *Lycodon ruhstrati* | KJ179951 | This study | III |
|  | *L. rufozonatum* | KJ179950 | This study | III-C |
|  | *L. flavozonatum* | KR911720 | This study | III-C |
|  | *L. semicarinatum^*,#^* | NC_001945 | Kumazawa et al., 1998 | III-C |
|  | *Euprepiophis perlacea* | NC_024546 | Wan et al., 2014, | III |
|  | *Orthriophis taeniurus* | NC_025275 | Li et al., 2014 | III-C |
|  | *Elaphe poryphyracea* | NC_012770 | Lin et al., 2012 | III |
|  | *E. bimaculata* | NC_024743 | Yan et al., 2014 | III-C |
|  | *E. anomala* | NC_027001 | Liu and Zhao, unpublished | III |
|  | *E. schrenckii* | KP888955 | Liu and Zhao, 2015 | III |
|  | *E. davidi* | NC_025643 | Xu, unpublished | III |
|  | *Oocatochus rufodorsatus* | NC_022146 | Li et al., 2013 | III-C |
|  | *Pantherophis slowinskii^*,#^* | NC_009769 | Jiang et al., 2007 | III-C |
|  | *P. guttatus* | AM236349 | Douglas et al., 2006 | III-C |
|  | *Nerodia sipedon* | JF964960 | Huff et al., 2011 | III |
|  | *Hebius vibakari* | KP684155 | Xu and Zhao, 2015 | III |
|  | *Thermophis zhaoermii* | NC_012816 | He et al., 2010 | III-C |
|  | *Hypsiglena torquata* | EU728591 | Mulcahy and Macey, 2009 | III |
|  | *H. sp.* | EU728580 | Mulcahy and Macey, 2009 | III |
|  | *H. slevini* | EU728584 | Mulcahy and Macey, 2009 | III |
|  | *H. ochrorhyncha ochrorhyncha* | EU728578 | Mulcahy and Macey, 2009 | III |
|  | *H. o. nuchalata* | EU728581 | Mulcahy and Macey, 2009 | III |
|  | *H. o. klauberi* | EU728589 | Mulcahy and Macey, 2009 | III |
|  | *H. jani* | EU728592 | Mulcahy and Macey, 2009 | III |
|  | *H. chlorophaea chlorophaea* | NC_013977 | Mulcahy and Macey, 2009 | III |
|  | *H. c. deserticola* | EU728587 | Mulcahy and Macey, 2009 | III |
|  | *H. c. catalinae* | KJ486459 | Mulcahy et al., 2014 | III |
|  | *H. unaocularus* | NC_024164 | Mulcahy et al., 2014 | III |
|  | *Pseudoleptodeira latifasciata* | NC_013981 | Mulcahy and Macey, 2009 | III |
|  | *Sibon nebulatus* | EU728583 | Mulcahy and Macey, 2009 | III-G |
|  | *Imantodes cenchoa* | EU728586 | Mulcahy and Macey, 2009 | III-E |
|  | *Leptodeira septentrionalis* | EU728590 | Mulcahy and Macey, 2009 | III-F |
| Outgroup |  |  |  |  |
| Iguanidae | *Iguana iguana* | AJ278511 | Janke et al., 2001 |  |
| Scincidae | *Plestiodon egregius* | NC_000888 | Kumazawa and Nishida, 1999 |  |
| Varanidae | *Varanus komodoensis* | AB080275/AB080276 | Kumazawa and Endo, 2004 |  |
