# Supplementary material for: Multiple independent structural dynamic events in the evolution of snake mitochondrial genomes
Source: BMC Genomics. 2018 May 10;19:354. doi: 10.1186/s12864-018-4717-7 (PMC5946542; doi:10.1186/s12864-018-4717-7)
Supplement: Supplementary file 6 — Table S3. Primers sequences used in this study. (DOC 66 kb) [file 12864_2018_4717_MOESM6_ESM.doc]

**Table S3 Primer sequences used in this study**

| No. of primer pair | Primer name | Primer sequences (5′→ 3′) | Length (bp) |
| --- | --- | --- | --- |
| 1 | L.1-F | TAAAGCATAGCACTGAAAATGCTAAG | 1088 |
|  | L.1-R | GTTGTTACTGTTCGATCACATCTAC |  |
| 2 | L.2-F | CGACATTACCATAATCTTTTTGAGC | 1061 |
|  | L.2-R | GAAGTTCTTTTTTTTTCCAAGGTCG |  |
| 3 | L.3-F | CATAAGACCAGAAGACCCTGTG | 1166 |
|  | L.3-R | ATGAATAGAATGTTGGTGTATTCTGC |  |
| 4 | L.4-F | TAGCCATAATATGATTCACATCCAC | 1553 |
|  | L.4-R | AAAGGAGATGATTTGAACATCTGTG |  |
| 5 | L.5-F | CCAATTATCTCAACAATCTCCCG | 1116 |
|  | L.5-R | AGGGGTGATAGTGTTATTGATGATA |  |
| 6 | L.6-F | TCCTATCAGGATTCATACCAAAATG | 1067 |
|  | L.6-R | AAATTAATTGCCCCCAGGATGGA |  |
| 7 | L.7-F | CAGGAAACCTAGTACACTCAGG | 1170 |
|  | L.7-R | GTCCCGTCTTTTCTCGGG |  |
| 8 | L.8-F | CATCGTATGAGAAGCACTAACATG | 1223 |
|  | L.8-R | AGGGCTGTTGGGATTATTATAGTTC |  |
| 9 | L.9-F | CCTCAGTGGTATGCCACAACT | 1093 |
|  | L.9-R | GCTCAGAAAAAGCCAAGGAAGAA |  |
| 10 | L.10-F | CGCATTATTCGAGAAAGCACCTA | 1109 |
|  | L.10-R | GCTATATTAGGTGTTTTTGTTGTAGTGA |  |
| 11 | L.11-F | GCTTTCGACCCAAAAGAACTTAC | 1123 |
|  | L.11-R | GGGTATGCGATAAGGGATTTTAG |  |
| 12 | L.12-F | GGCATTATTCGAATAACACAAATCCT | 1352 |
|  | L.12-R | GTAAGTTCTTGTATATTGATTGATGATG |  |
| 13 | L.13-F | CTTATCGGGTGATGACACGG | 1155 |
|  | L.13-R | GGGTTCACAGGTCTATTAGTTC |  |
| 14 | L.14-F | CCATCATTGGAGTTTTACTATCAAAAGA | 1337 |
|  | L.14-R | GCAGAATGATATTTGTCCTCATGG |  |
| 15 | L.15-F | CCTACGGATGAATCATACAAAACC | 979 |
|  | L.15-R | GGGGGGCTAGAGATGGG |  |
| 16 | L.16-F | CAGTAGACCCCCATTCACC | 1465 |
|  | L.16-R | CTAATATTAACTACTGCTGCGGG |  |
| 17 | L.1/2-F | GCGTACTGGAAAGTGCGCT | 303 |
|  | L.1/2-R | TGGTTTCGGGGTGAAGGC |  |
| 18 | L.4/5-F | TTCCGCTACGACCAATTAATACAC | 2098 |
|  | L.4/5-R | ATATGTTCCTAGTCTTTGATTGTTATTGA |  |
| 19 | L.6/7-F | GCCCAGCATTTATTATAATTTTCTTCAT | 485 |
|  | L.6/7-R | CAAAGAATCAGAAAGGTGTTGGAATA |  |
