# Supplementary material for: Multiple independent structural dynamic events in the evolution of snake mitochondrial genomes
Source: BMC Genomics. 2018 May 10;19:354. doi: 10.1186/s12864-018-4717-7 (PMC5946542; doi:10.1186/s12864-018-4717-7)
Supplement: Supplementary file 7 — Table S4. Best-fit models and partitioning schemes selected by PartitionFinder for the dataset analyzed. (DOCX 22 kb) [file 12864_2018_4717_MOESM7_ESM.docx]

Table S4 Best-fit models and partitioning schemes selected by PartitionFinder

| **Dataset - MrBayes** | | |
| --- | --- | --- |
| Partition | Subsets included | Substitution model |
|  | *tRNA-Gly*, *tRNA-Ile*, *tRNA-Trp* | GTR+G |
|  | *ND4L* position 2, *tRNA-Leu(UUR)* | GTR+G |
|  | *tRNA-Pro*, *tRNA-Gln*, *tRNA-Tyr* | GTR+G |
|  | *tRNA-Met*, *COX3* position 2, *COX2* position 1 | GTR+I+G |
|  | *tRNA-Asn*, *tRNA-Ala* | GTR+G |
|  | *tRNA-Cys* | SYM+G |
|  | *tRNA-Ser(UCN)* | GTR+G |
|  | *tRNA-Asp*, *ND4L* position 1 | GTR+G |
|  | *tRNA-Lys*, *tRNA-Leu(CUN)* | GTR+I+G |
|  | *tRNA-Arg* | SYM+G |
|  | *tRNA-His*, *12s RNA*, *tRNA-Val* | GTR+I+G |
|  | *ND1* position 2, *tRNA-Th*r, *tRNA-Ser(AGY)*, *tRNA-Phe* | GTR+I+G |
|  | *tRNA-Glu* | SYM+G |
|  | *16s RNA* | GTR+I+G |
|  | *ND5* position 3, *ATP6* position 1 | GTR+I+G |
|  | *Cyt b* position 1, *ATP6* position 2 | GTR+I+G |
|  | *ATP6* position 3 | GTR+G |
|  | *ATP8* position 1, *ATP8* position 3 | GTR+I+G |
|  | *ATP8* position 2 | HKY+I+G |
|  | *COX1* position 1 | GTR+I+G |
|  | *COX1* position 2 | SYM+I+G |
|  | *COX1* position 3 | HKY+I+G |
|  | *COX2* position 2 | GTR+I+G |
|  | *COX2* position 3, *ND4L* position 3 | GTR+I+G |
|  | *COX3* position 1 | GTR+I+G |
|  | *Cyt b* position 2, *COX3* position 3 | GTR+I+G |
|  | *Cyt* b position 3 | GTR+G |
|  | *ND*1 position 1 | GTR+I+G |
|  | *ND2* position 3, *ND1* position 3 | GTR+I+G |
|  | *ND2* position 1 | GTR+I+G |
|  | *ND2* position 2 | GTR+I+G |
|  | *ND3* position 1 | GTR+I+G |
|  | *ND3* position 2 | GTR+I+G |
|  | *ND3* position 3 | GTR+I+G |
|  | *ND4* position 1 | GTR+I+G |
|  | *ND4* position 2 | GTR+I+G |
|  | *ND4* position 3 | GTR+I+G |
|  | *ND5* position 1 | GTR+G |
|  | *ND5* position 2 | GTR+G |

| **Dataset - RAxML** | | |
| --- | --- | --- |
| Partition | Subsets included | Substitution model |
|  | *tRNA-Trp*, *tRNA-Ile* | GTR+I+G |
|  | *tRNA-Leu(UUR)*, *ND4L* position 2 | GTR+I+G |
|  | *tRNA-Pro*, *tRNA-Tyr*, *tRNA-Gln* | GTR+I+G |
|  | *tRNA-Met*, *COX3* position 2, *COX2* position 1 | GTR+I+G |
|  | *tRNA-Asn*, *tRNA-Ala* | GTR+I+G |
|  | *tRNA-Cys* | GTR+I+G |
|  | *tRNA-Ser(UCN)* | GTR+I+G |
|  | *tRNA-Asp*, *ND4L* position 1 | GTR+I+G |
|  | *tRNA-Lys*, *tRNA-Leu(CUN)* | GTR+I+G |
|  | *tRNA-Gly* | GTR+I+G |
|  | *tRNA-Arg* | GTR+I+G |
|  | *tRNA-His*, *12s RNA*, *tRNA-Val* | GTR+I+G |
|  | *ND1* position 2, *tRNA-Thr*, *tRNA-Phe*, *tRNA-Ser(AGY)* | GTR+I+G |
|  | *tRNA-Glu* | GTR+I+G |
|  | *16s RNA* | GTR+I+G |
|  | *ND5* position 3, *ATP6* position 1 | GTR+I+G |
|  | *Cyt b* position 1, *ATP6* position 2 | GTR+I+G |
|  | *ATP6* position 3 | GTR+I+G |
|  | *ATP8* position 3, *ATP8* position 1 | GTR+I+G |
|  | *ATP8* position 2 | GTR+I+G |
|  | *COX1* position 1 | GTR+I+G |
|  | *COX1* position 2 | GTR+I+G |
|  | *COX1* position 3 | GTR+I+G |
|  | *COX2* position 2 | GTR+I+G |
|  | *COX2* position 3, *ND4L* position 3 | GTR+I+G |
|  | *COX3* position 1 | GTR+I+G |
|  | *Cyt b* position 2, *COX3* position 3 | GTR+I+G |
|  | *Cyt b* position 3 | GTR+I+G |
|  | *ND1* position 1 | GTR+I+G |
|  | *ND2* position 3, *ND1* position 3 | GTR+I+G |
|  | *ND2* position 1 | GTR+I+G |
|  | *ND2* position 2 | GTR+I+G |
|  | *ND3* position 1 | GTR+I+G |
|  | *ND3* position 2 | GTR+I+G |
|  | *ND3* position 3 | GTR+I+G |
|  | *ND4* position 1 | GTR+I+G |
|  | *ND4* position 2 | GTR+I+G |
|  | *ND4* position 3 | GTR+I+G |
|  | *ND5* position 1 | GTR+I+G |
|  | *ND5* position 2 | GTR+I+G |
